# Correlations between Integrin ανβ6 Expression and Clinico-Pathological Features in Stage B and Stage C Rectal Cancer

**DOI:** 10.1371/journal.pone.0097248

**Published:** 2014-05-12

**Authors:** Seong Beom Ahn, Abidali Mohamedali, Charles Chan, Julie Fletcher, Sun Young Kwun, Candice Clarke, Owen F. Dent, Pierre H. Chapuis, Edouard Nice, Mark S. Baker

**Affiliations:** 1 Australian School of Advanced Medicine, Macquarie University, North Ryde, New South Wales, Australia; 2 Department of Chemistry and Biomolecular Sciences, Macquarie University, North Ryde, New South Wales, Australia; 3 Department of Anatomical Pathology, Concord Hospital, University of Sydney, Sydney, New South Wales, Australia; 4 Department of Colorectal Surgery, Concord Hospital, University of Sydney, Sydney, New South Wales, Australia; 5 Department of Biochemistry and Molecular Biology, Monash University, Clayton, Victoria, Australia; University of Texas MD Anderson Cancer Center, United States of America

## Abstract

Integrin ανβ6 is highly expressed in a range of human cancers and frequently correlates with patient survival. This study examines correlations between ανβ6 expression and patient clinico-pathological features in Stage B and Stage C rectal cancer, including overall survival. Expression of ανβ6 was measured in 362 Stage B or C rectal cancer tissue samples at the tumour central region, invasive tumour front and adjacent non-neoplastic mucosa using immunohistochemistry. Distribution of ανβ6 was found to be significantly higher at the invasive front compared to central regions of the tumour (p<0.001) or adjacent non-neoplastic mucosa (p<0.001) suggesting ανβ6 plays a role in tumour cell invasion. However, integrin ανβ6 expression was not associated with clinico-pathological features or overall survival indicating it is not an independent prognostic marker differentiating Stage B or C rectal cancer. Previous ανβ6 studies have suggested the expression of ανβ6 is involved in the earlier stages (i.e. Stages A/B) of tumour progression rather than the later stages (i.e. Stages C/D). However, our study has revealed that in rectal cancer ανβ6 expression does not increase between Stages B and C, but may occur earlier, namely before or during Stage B cancer.

## Introduction

Classification of the severity of colorectal cancer (CRC) is currently based on clinical and histological determination of extent of spread of the tumour. Tumour cells can be localised within the bowel wall (Stage A), extending beyond the muscularis propria (Stage B), or metastasised to lymph nodes (Stage C) or distant organs (Stage D). Five-year CRC survival statistics show a significant reduction in survival between Stage B and C, corresponding to difference in lymph node metastasis [1]. Although clinico-pathological staging is the current gold standard technique for the determination of prognosis, the use of new protein biomarkers expressed or amplified in a Stage-specific manner during progression may increase the precision and reliability of histological determinations. A combination of a sound CRC staging system and presence of prognostic protein biomarkers has been proposed as a more efficacious approach for diagnosis, prognosis and treatment guidance [2].

One recently proposed potential prognostic biomarker is the integrin ανβ6. This protein is highly expressed in many types of cancers and has been suggested to be a prognostic indicator of poor survival in CRC, gastric adenocarcinoma and cervical squamous carcinoma [3]–[6]. Integrin ανβ6 is a transmembrane receptor composed of non-covalently linked αν and β6 subunits, where the β6 subunit partners exclusively with αν and is expressed only in epithelial tissues [7]. ανβ6 is often concentrated in poorly differentiated tumours proximal to invading cancer margins [8]–[11]. It has also been identified as an important factor in the *“epithelial to mesenchymal transition”* (EMT) that is characterised by loss of cell adhesion, repression of E-cadherin and increased cell motility during carcinoma progression [12]. Integrin ανβ6 is thought to operate in a complex manner, initiating cell signalling cascades whilst also interacting with key extracellular matrix (ECM) proteins and activating growth signals such as latent transforming growth factor β1 (TGF-β1), a recognised inducer of EMT [3], [13]. In support of this contention, antibody-mediated inhibition of ανβ6-mediated TGF-β1 activation suppresses EMT [14]. In addition, ανβ6 integrin has been shown to play a vital role in a CRC spheroid EMT model mediated through TGF-β1 activation and subsequent migration of cells on interstitial fibronectin [11].

In contrast to most other integrins, ανβ6 signals through a unique 12-mer C-terminal cytoplasmic sequence that directly interacts with the extracellular signal-regulated kinase (ERK2) activating the ERK/MAPK pathway that is often highly overexpressed in CRC metastasis [15]. The β6•ERK2 interaction is also responsible for integrin-mediated matrix metalloprotease 9 (MMP9) secretion (through MAPK) that allows degradation of ECM, facilitating cell escape [16], [17]. In summary, ανβ6 is a regulator of metastasis and is found to be overexpressed in many cancer phenotypes. Whilst the body of evidence implicating ανβ6 in metastasis is substantial, currently no studies have examined when it becomes overexpressed or whether correlation exists between ανβ6 and patient survival in rectal cancer. In this study, we specifically examine immunohistochemical expression of ανβ6 in 362 patients with rectal cancer Stage B or C in the central region of the tumour, the invasive front and adjacent non-neoplastic mucosa.

## Materials and Methods

### Patient cohort

All patients underwent surgical resection for rectal cancer at Concord Hospital, a tertiary referral hospital in Sydney, Australia, between January 1988 and December 2001. All resections were performed by specialist colorectal surgeons following a standardised technique (total anatomical dissection) [18]. The rectum was defined as including the rectosigmoid junction but excluding the anal canal. Clinical data from the patients were entered into a prospective database initiated in 1971, including information on patient characteristics, comorbidity, presentation, investigations, surgical management, complications, adjuvant therapy, pathology and follow-up [19], [20]. The CRC Project at Concord Hospital is carried out under the approval of the South Western Sydney Health Area Ethics Committee (CH62/6/2011-136) with written consents in accordance with the requirements of the NSW Human Tissue Act 1983 and the NHMRC National Statement on Ethical Conduct in Human Research 2007. The study also approved by the Macquarie University Human Ethics Committee (#5201100858). Patients who received neoadjuvant radiotherapy had either short or long course treatment with or without associated chemotherapy. Selection for treatment was based on clinical findings and discussion in a multidiscipline meeting.

### Pathological examination of the resected specimen

Examination of resected specimens followed a standard protocol [1]. Tumour size was measured as the greatest surface dimension. Blocks were taken to demonstrate maximum direct tumour penetration of the bowel wall. Additional blocks were taken specifically to demonstrate the relationship between tumour and any adherent structure or tissue [21] as well as lines of resection and the free serosal surface [22]. Tumour level in the rectum was measured from the anal verge. Venous invasion by the tumour referred to involvement of thick or thin walled veins, either within or beyond the bowel wall. When doubt existed as to whether a structure involved was a vein, a negative finding was recorded. An apical node was defined as the most proximal of any nodes found within 1cm of the ligation of a named vessel at the apex of a vascular pedicle. Tumour grade was assessed taking into account the degree of differentiation and anaplasia, the nature of the tumour margin (pushing or infiltrating) and the presence and prominence of vascular invasion [19]. All pathological characteristics were analysed in every specimen and presence or absence recorded explicitly, with no missing data on any variable.

Tumours were staged according to the Australian Clinico-Pathological Staging (ACPS) system [1] for CRC which accommodates sub-stages compatible with other clinicopathologic staging systems such as TNM [23]. A potentially curative operation was defined as one where there were no systemic metastases at time of operation and no tumour identified histologically in the proximal, distal or circumferential lines of resection histologically (ACPS Stages A, B, C).

### Tissue microarray construction

Tissue microarrays (TMA) were constructed using an Advanced Tissue Arrayer ATA-100 (Chemicon, Temecula, CA, USA). Cores (1.5 mm) were taken from carefully selected, morphologically representative areas of the original paraffin blocks and arrayed into freshly made recipient paraffin blocks. Cores were taken from the central region of the tumour (avoiding luminal surfaces), the invasive front of the tumour and histologically normal mucosa.

### Immunohistochemistry (IHC)

A murine monoclonal antibody (MAb) against full length human ανβ6 (clone 6.2A1, IgG1) (Biogen Idec, Cambridge, MA, USA) was used in IHC. The specificity of the anti-ανβ6 monoclonal antibody 6.2A1 has been reported in several studies [14], [24]–[26]. All TMA sections were prepared and processed simultaneously with the same batch of primary and secondary antibodies and staining reagents, obviating the need to deploy an internal standard. IHC was performed with two different detection amplification systems: an avidin-biotin complex (ABC) and a polymer-based IHC detection system. For ABC IHC, 4 µm paraffin-embedded TMA sections were deparaffinised and rehydrated, and endogenous peroxidase blocked with 3% H_2_O_2_ in methanol for 5min. Antigen retrieval was performed using pepsin (DAKO, Carpinteria, CA) at 37°C for 10min. Sections were incubated with horse serum (Vector Laboratories, Burlingame, CA) blocking solution (15 µl/ml) in TBS-Tween20 for 20min at room temperature (RT) and then incubated with 0.5 mg/ml anti-ανβ6 MAb at RT for 60min. Sections were incubated with biotinylated anti-mouse IgG (Vector Laboratories) for 20min at RT and incubated with Elite ABC reagent (Vector Laboratories) for 20min at RT. Between each incubation, TBS washing was performed twice. 3,3'-diaminobenzidine substrate in 0.05 M Tris-HCL (pH 7.4) was applied for 5min and intensity enhanced by incubating sections in CuSO_4_ for 5min. Cell nuclei were counterstained with Mayer's Hematoxylin (Sigma, St. Louis, MO). Polymer-based IHC performed on a Bond-Max Autostainer (Leica Microsystems, Bannockburn, IL) as described [27], except that antigen retrieval was performed with pepsin and 6.2A1 anti-ανβ6 MAb (0.5 µg/ml) used as a primary antibody. Isotype IgG1 (R&D Systems, Minneapolis, USA) was used as a negative control.

### IHC evaluation

Immunoreactivity for ανβ6 was evaluated by two assessors independently (SBA, CC), with 100% agreement, who were blinded to patient's clinico-pathological status. Staining intensities were scored separately for central region, invasive tumour front and normal mucosa and scored as 0 = no staining, 1 = weak staining, 2 = intermediate staining and 3 = strong staining. If staining intensity was heterogeneous in any single tissue core, the predominant staining intensity was recorded.

### Outcome variable and patient follow-up

The outcome of overall survival time was measured from date of surgical resection to date of death due to any cause, with times censored for patients lost to follow-up or who remained alive at the close of the study. Patients were followed annually until death or to December 31, 2011. Details of the follow-up protocol have been described previously [18].

### Statistical Analysis

The chi-squared test or Fisher's exact test were used to examine the statistical significance of differences in proportions. The Wilcoxon matched pairs signed ranks test was used to compare the frequency distributions of ανβ6 expression between the central tumour, frontal tumour and normal mucosa. Comparisons of survival time between strata of ανβ6 expression and covariates were made with the Kaplan-Meier method and log-rank test and also Cox regression and Wald p. Continuous and multi-category covariates were dichotomised at conventional or otherwise appropriate cutting points. As clinico-pathological stage is the strongest known predictor of prognosis, associations with survival were examined for Stage B and C separately as well as for the two stages combined, in order to identify any differences in effects of ανβ6 between stages. The level for two-tailed statistical significance was p≤0.05 with confidence intervals (CI) at the 95% level. Analyses were performed with SPSS version 20 (IBM Australia Limited).

## Results

From the 1,804 Concord Hospital CRC resections between January 1988 and December 2001, patients were excluded in the following sequence: those patients with colon cancer (1,022), previous CRC (20), inflammatory bowel disease or *polyposis coli* (9), a first degree relative with CRC (65) and Stage A or D tumour (289), leaving 399 patients potentially available for assessment of ανβ6 in this study. However, 37 of these patients had insufficient archival tissue available for assessment, leaving 362 who were assessable. Varying numbers of these had an uninformative result for one or more of the ανβ6 assessments (indicated where appropriate in subsequent tables). On comparing the 362 patients who had a result on one or more of the ανβ6 assessments with the 37 who could not be assessed, there were no material differences over the range of clinico-pathological variables examined. Thus the 362 patients assessed appeared to be representative of the total pool of 399 patients from which they were drawn. The clinico-pathological characteristics of these 362 patients are shown in Table 1.

**Table 1:** Clinico-pathological features of 362 patients for whom data were available for at least one of the measures of integrin ανβ6 expression.

Variable	Category	Number (%) or Median (range)
Sex	Male	235 (65)
	Female	127 (35)
Age (years)		67 (29–94)
Tumour distance from anal verge (cm)		10.2 (2–19)
Type of resection	Abdominoperineal excision	64 (18)
	Hartmann's operation	24 (7)
	Restorative or other operation	274 (76)
Tumour maximum surface dimension (cm)		5.0 (1–19)
Distal clearance margin (cm)		4.1 (0.01–17)
Histological type of tumour	Adenocarcinoma	339 (94)
	Mucinous adenocarcinoma	21 (5)
	Signet ring adenocarcinoma	2 (1)
Direct tumour spread	Confined to submucosa	7 (2)
	Not beyond muscularis propria	24 (7)
	Beyond muscularis propria	331 (91)
Number of nodes involved	None (N0)	168 (46)
	1–3 nodes (N1)	122 (34)
	> 3 nodes (N2)	72 (20)
Tumour stage	Stage B	168 (46)
	Stage C	194 (54)
Tumour grade	Low	20 (6)
	Average	244 (67)
	High	98 (27)
Venous invasion	None	260 (72)
	Mural	13 (4)
	Extra-mural	69 (19)
	Both	20 (6)
Free serosal surface involved	No	342 (95)
	Yes	20 (6)
Adjacent organ or structure infiltrated	No	353 (98)
	Yes	9 (2)
Preoperative radiotherapy with or without chemotherapy	No	344 (95)
	Yes	18 (5)
Postoperative radiotherapy	No	343 (95)
	Yes	19 (5)
Postoperative chemotherapy	No	305 (84)
	Yes	57 (16)

### Expression of integrin ανβ6 in Stage B and C rectal cancer tissue samples

**Figure 1:**
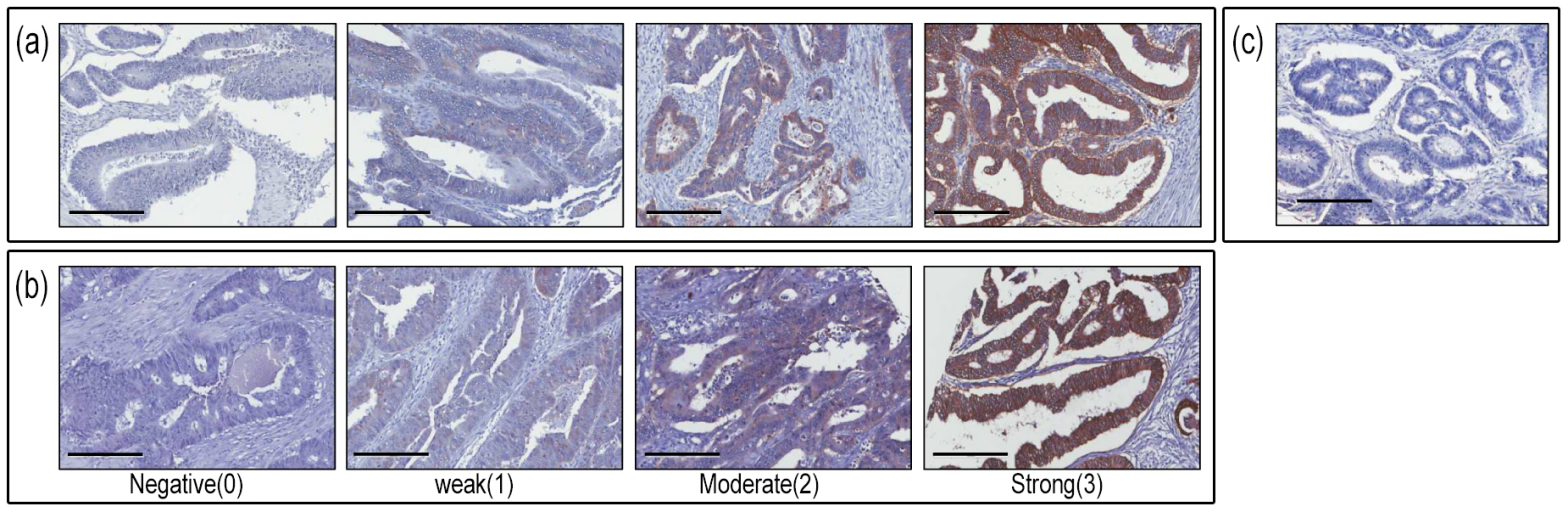
Integrin ανβ6 staining intensity in rectal cancer Stage B (a) and C (b). There was no difference in staining patterns within each scored value (0 or 1 or 2 or 3) between rectal cancer Stage B and C tissues. (c): IgG1 negative control. Scale bar: 200 µm.

In order to compare distribution of ανβ6 staining intensity between central, frontal rectal cancer and histologically normal epithelial tissue, and because of the varying numbers of patients with informative results on each assessment, a Wilcoxon matched pairs signed ranks test was performed in patients who had an informative result on both assessments of each pair. The distribution of ανβ6 staining intensity was significantly higher in the frontal compared to central tissue (n = 259, p<0.001); significantly higher in the frontal compared to normal tissue (n = 302, p<0.001); and slightly higher in the central compared to apparently normal mucosa (n = 253, p = 0.049) (Table 2). The intensities of ανβ6 expression between the rectal cancer Stage B and C tissues showed no difference within each scored value (Figure 1). Non-specific binding was examined using relevant isotype control MAbs and was shown in all cases to be inconsequential.

**Table 2:** Distribution of ανβ6 expression in central and frontal tumour tissue and in adjacent non-neoplastic mucosa and. Number (%).

ανβ6 assessment	Number of patients	Negative	Weak	Intermediate	Strong
Central tumour tissue	277	61 (22)	66 (24)	117 (42)	33 (12)
Frontal tumour tissue	341	47 (14)	58 (17)	156 (46)	80 (24)
Adjacent non-neoplastic mucosa	322	80 (25)	84 (26)	145 (45)	13 (4)

### Follow up detail

In 110 (30.4%) patients who had not died, survival time ranged from 103 months to 245 months with a median of 171 months. In 243 (67.1%) patients who had died, survival time ranged from 2 days to 243 months with a median of 45 months. In 9 (2.5%) patients who were lost, follow-up time ranged from 3 to 178 months with a median of 51 months.

### Expression of ανβ6 and patients survival

**Figure 2:**
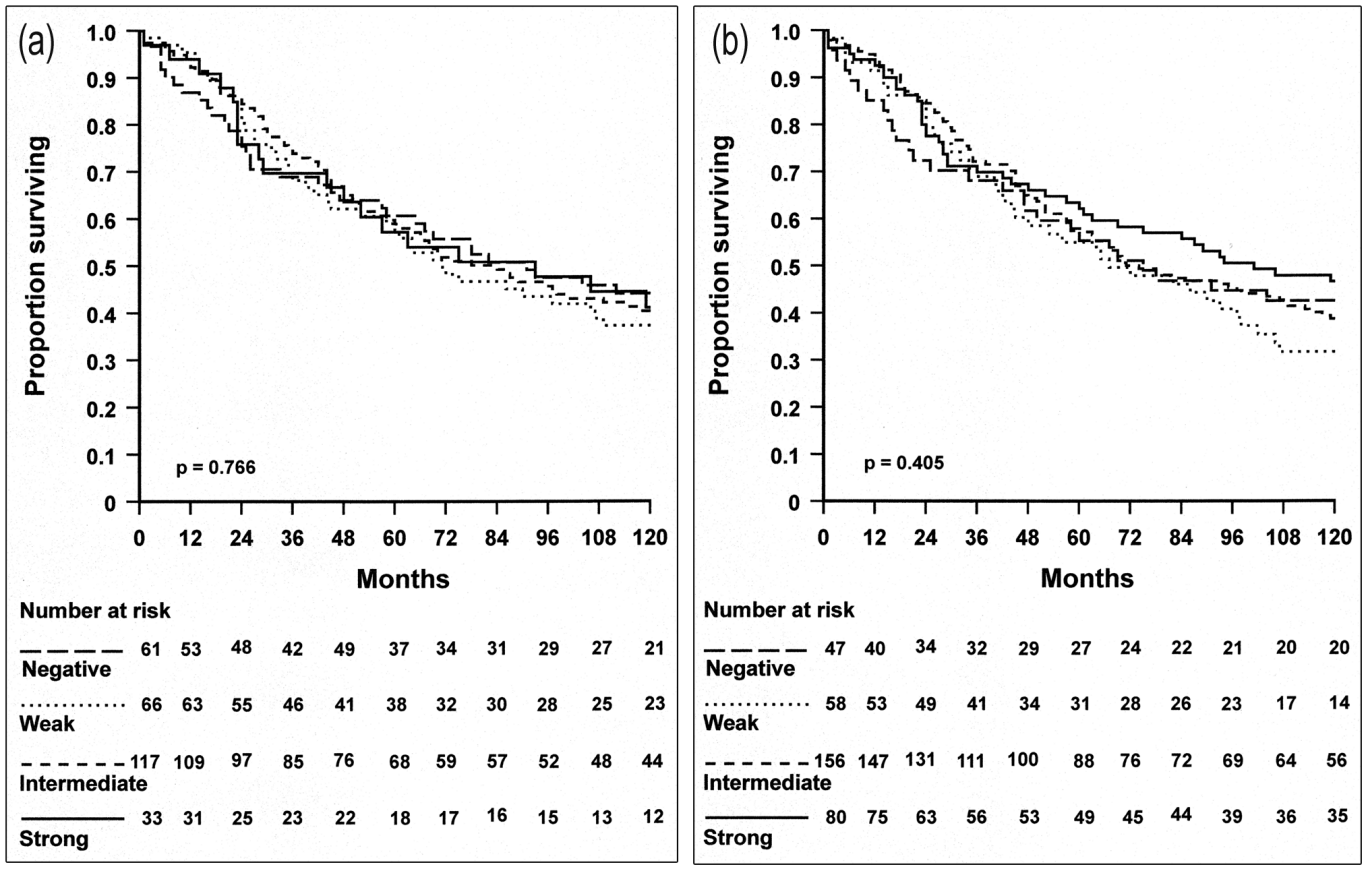
Kaplan-Meier survival analysis using a Log-rank test. Overall survival of 362 rectal cancer patients (combined Stage B and C) was not significantly related to central (a) or frontal (b) ανβ6 expression.

Kaplan-Meier survival plots demonstrated that overall survival was not significantly related to either central or frontal ανβ6 expression, either for Stage B and C combined or separately (Figure 2 and Table 3).

**Table 3:** Association between integrin ανβ6 central and frontal expression in Stage B and C tumours.

ανβ6 site	Stage	Number of patients	Negative	Weak	Intermediate	Strong	Chi^2^ for B vs. C	Hazard ratio (95%CI)	Wald p
Central	Stage B & C	277	61	66	117	33	0.432	1.0 (0.9–1.2)	0.660
	Stage B	125	30	34	47	14	—	1.0 (0.8–1.2)	0.709
	Stage C	152	31	32	70	19	—	1.1 (0.9–1.3)	0.525
Frontal	Stage B & C	341	47	58	156	43	0.736	1.0 (0.8–1.1)	0.503
	Stage B	165	26	29	73	37	—	1.0 (0.8–1.2)	0.808
	Stage C	176	21	29	83	43	—	0.9 (0.8–1.1)	0.370

Association between overall survival and ανβ6 central and frontal expression in Stage B and C combined and in Stage B and C separately.

### Expression of ανβ6 and clinico-pathological features

The assessment of correlation between integrin ανβ6 expression and clinico-pathological features indicated that ανβ6 expression was not associated with any clinico-pathological feature (Table 3).

### Clinico-pathological features of Stage B and C rectal cancer patient

Overall survival was significantly diminished in patients aged 75 years or older, those who had a Hartmann's operation, those with mucinous or signet ring tumours, when an apical lymph node was involved, when ≥4 nodes were involved, those with Stage C tumour, in high grade tumours, in the presence of venous invasion, when an adjacent organ or structure had been infiltrated by tumour, and in patients who did not receive adjuvant chemotherapy. Association between clinico-pathological features and overall survival in the 362 patients is presented in Table 4.

**Table 4:** Association between clinico-pathological features and overall survival in 362 patients for whom data were available on at least one of the measures of ανβ6 expression.

Variable	Patients	Deaths	Hazard ratio (95% CI)	Wald p
Male	235	154	0.9 (0.7–1.2	0.693
Female	127	89		
Age ≥ 75 years	98	82	1.8 (1.4–2.4)	<0.001
No	264	161		
Tumour level ≤ 6 cm	84	51	0.8 (0.6–1.1)	0.211
No	278	192		
Hartmann's operation	24	23	2.7 (1.7–4.1	<0.001
No	338	220		
Tumour size ≥ 5 cm	178	116	0.9 (0.7–1.2)	0.676
No	184	127		
Distal margin < 2 cm	37	27	1.1 (0.7–1.6)	0.696
No	325	216		
Spread past MP	331	223	1.2 (0.8–1.9)	0.463
No	31	20		
Mucinous, signet ring	23	15	1.5 (1.2–2.0)	0.003
No	339	228		
Apical node involved	12	10	2.8 (1.5–5.3)	0.001
No	350	233		
≥ 4 nodes involved	72	57	1.8 (1.3–2.4)	<0.001
No	290	186		
Stage C	194	141	1.5 (1.2–2.0)	<0.001
Stage B	168	102		
High grade	98	70	1.5 (1.2–2.0)	0.003
No	264	173		
Venous invasion	102	78	1.7 (1.3–2.2)	<0.001
No	260	165		
To free serosal surface	20	13	1.1 (0.6–2.0)	0.663
No	342	230		
To adjacent organ or structure	9	9	3.0 (1.5–5.8)	0.001
No	353	234		
Preoperative radio/chemotherapy	18	16	1.5 (0.9–2.6)	0.090
No	344	227		
Postoperative radiotherapy	19	15	1.4 (0.8–2.3)	0.252
No	343	228		
Postoperative chemotherapy	57	29	0.7 (0.5–0.99)	0.042
No	305	214		

### Detection of ανβ6 using polymer-based IHC

Polymer-based IHC was performed soley to detect ανβ6 missed by ABC IHC test. This was an investigative tool to determine only ανβ6 staining intensity and was not used to gauge patient survival. A comparison of distributions of integrin ανβ6 staining intensities of the two IHC techniques demonstrated that ανβ6 expression using the polymer based IHC occurred in more than 90% of all patients' tissues in all assessments (Table 5).

**Table 5:** A comparison of distribution of integrin ανβ6 staining intensities between polymer-based and avidin-biotin complex (ABC) IHCs in adjacent non-neoplastic mucosa and in central and frontal tumour tissue.

	Central tumour tissue		Frontal tumour tissue		Adjacent non-neoplastic mucosa	
	Polymer based IHC (n = 274)	ABC IHC (n = 274)	Polymer based IHC (n = 337)	ABC IHC (n = 337)	Polymer based IHC (n = 319)	ABC IHC (n = 319)
None	23 (8.4)	60 (21.9)	16 (4.7)	45 (13.4)	3 (0.9)	79 (24.8)
Weak	26 (9.5)	65 (23.7)	12 (3.6)	58 (17.2)	16 (5.0)	84 (26.3)
Intermediate	126 (46.0)	116 (42.3)	131 (38.9)	155 (46.0)	133 (41.7)	143 (44.8)
Strong	99 (36.1)	33 (12.0)	178 (52.8)	79 (23.4)	167 (52.4)	13 (4.1)

Number (%).

## Discussion

In this study, we demonstrate that distribution of integrin ανβ6 expression was significantly higher in the invasive front of the tumour compared to the central region of the tumour (p<0.001) or histologically normal mucosa (p<0.001) in 362 rectal cancers Stage B and C. However, clinico-pathological features and overall survival were not statistically associated with integrin ανβ6 expression. This observation contrasts with previous reports suggesting that ανβ6 expression is often associated with poor survival in various cancers [3], [6], [11], [26], [28]. However, the other ανβ6 studies were carried out on tumours across multiple Stages (i.e., Stages A to D) whereas our study focused only on Stage B and C and for the first time exclusively on rectal cancer samples.

CRC is a devastating disease whose protein molecular biosignatures are slowly being explored. An understanding and detection of these may help timely diagnosis, prognosis and treatment of CRC [2]. It is well known that metastasis is the major cause of mortality and a leading cause of the failure of anti-cancer therapies [29]. Indeed, it is established that a phenotypic histologically observable shift from a non-metastatic (Stage B) to nodal metastatic (Stage C) is correlated with poor survival [1]. At a molecular level, the switch of cancer cells from an epithelial to a mesenchymal phenotype is coupled with degradation of ECM and other pivotal biological processes that facilitate metastasis. Key regulators that may modulate these biological processes are loss of E-cadherin, activation of TGF-β1, increases in proteolytic systems, like MMPs, urokinase plasminogen activator (uPA) and the uPA receptor (uPAR) [13], [30]. Importantly, integrin ανβ6 is strongly associated with many of the processes these regulators act upon, including the MAPK pathway, one of the primary signalling pathways implicated in transformation, proliferation, invasion and metastasis of CRC [3], [31]. For example, ανβ6 activates TGF-β1 by releasing this protein from an inactive complex [3]. It is not surprising therefore, that ανβ6 is overexpressed primarily in proliferating epithelial cells where it activates and promotes “drivers” of metastasis. Our recent unpublished work suggest that ανβ6 interacts with other proteins, found up-regulated in CRC (e.g., uPAR), and this has now been confirmed by proximity ligation assay and peptide array. It is not known if interaction with other molecules may modulate the biology of ανβ6 given that uPAR is a multi-functional cell surface receptor involved in both ECM degradation and cellular signalling [32], as well as being a poor prognostic factor of CRC survival [33].

Not only do our results recapitulate previous studies demonstrating ανβ6 expression in proliferating epithelial cells, but they confirm that ανβ6 is epithelial-restricted in rectal cancer. Previous studies demonstrated that ανβ6 expression was concentrated at the invading edge of ovarian tumour and oral squamous cell carcinomas [8], [9], [34]. Our results confirm that ανβ6 is also more highly expressed at the invasive front in rectal cancer than in central regions of rectal tumours and/or normal mucosa suggesting ανβ6 plays crucial roles in tumour cell invasion.

This study focussed on delineating if in rectal cancer expression of ανβ6 could be a marker of a tumour's transition from Stage B to Stage C. Two IHC staining protocols were undertaken and the data from ABC-based IHC confirmed that integrin ανβ6 was not an independent prognostic marker in these two stages, nor was it correlated with any clinico-pathological feature studied. This result was contrary to expectations because ανβ6 has previously had a positive correlation with patient survival in a range of cancers. Specifically, ανβ6 has been identified as a prognostic indicator of poor survival in CRC [11], gastric adenocarcinoma [6], [26] and cervical squamous carcinoma [28], where the CRC and gastric carcinoma studies were based on tumour Stage I through IV and the cervical squamous carcinoma was on patients identified as FIGO Stage IA through IIB (equivalent to TMN Stage I–II). From that CRC study (n = 488) [11], Kaplan-Meier plots demonstrated that ανβ6 was strongly associated with survival in early stage tumours (Stage I–II) but not for the later stage tumours (Stage III–IV). Similarly, a study of 300 gastric carcinoma patients [26] revealed that ανβ6 was a potential risk factor in both early (Stage I–II) and late stage (Stage III–IV), with survival more significantly associated in earlier than later stage. A recent gastric carcinoma study (n = 51) [6] also demonstrated ανβ6 was a poor prognostic factor but survival was not significantly associated with stage. Moreover, the cervical study (n = 85) [28] indicated ανβ6 is an unfavourable prognostic factor in patients between FIGO Stage IA2-IB1 and IB2-IIB. Overall, the expression of ανβ6 is suggested to be involved in the earlier stages of tumour progression rather than the later stages, however our data has reveals that the ανβ6 expression does not increase between rectal cancer Stages B and C, but may occur earlier.

In addition, comparison of two alternative staining protocols (polymer-based versus an ABC IHC method) showed the more sensitive polymer-based amplification method detected ανβ6 expression in almost all (>90%) rectal cancer Stage B and C tissues. These data also supports that the ανβ6 expression may occur earlier than anticiptaed in CRC progression, namely before or during Stage B cancer.

Previous studies found ανβ6 expression is low or undetectable in normal adult epithelia, but is highly expressed during wound healing and/or cancer [3], [8], [34], potentially explaining why ανβ6 is being explored as an interesting target for cancer imaging and therapy [35]. Here, ανβ6 was observed in almost all histological normal rectal mucosa less than 1–2 cm from the tumour margin (i.e., adjacent non-neoplastic mucosa, suggesting that EMT-associated changes are occurring in that tissue). The observation that apparently adjacent non-neoplastic mucosa expresses other antigens involved in cancer progression (e.g., EMT) is supported by a study demonstrating EMT markers (α-smooth muscle actin & SNAIL) and EMT-inducers (MMP2 & TGF-β3) are extensively expressed in histologically normal tissues proximal to breast tumour margins (i.e.,  = 1 cm away) whilst being only sparsely expressed at a distance of 5 cm from the same tumour margins [36]. This study closely aligns with our current observations of ανβ6 being expressed within 1 cm of rectal cancer margins, as ανβ6 is also known to be involved in the EMT [14].

In conclusion, integrin ανβ6 is more frequently expressed at the invasive front of rectal cancer. While it likely plays an important role in tumour progression, this integrin does not act as an independent prognostic marker in rectal cancer Stage B and C. Further studies are needed to delineate proteins differentially expressed in different stages of rectal cancer.
