# Correction: Correlations between Integrin ανβ6 Expression and Clinico-Pathological Features in Stage B and Stage C Rectal Cancer

**DOI:** 10.1371/journal.pone.0104724

**Published:** 2014-08-01

**Authors:** The PLOS ONE Staff

In the fifth section of the “Immunohistochemistry (IHC)” section of the Materials and Methods, there is an error in the units. The corrected sentence should read: “Sections were incubated with horse serum (Vector Laboratories, Burlingame, CA) blocking solution (15 µl/ml) in TBS-Tween20 for 20min at room temperature (RT) and then incubated with 0.5 µg/ml anti-ανβ6 MAb at RT for 60min.”
